# Supplementary material for: AffyMAPSDetector: a software tool to characterize Affymetrix GeneChip™ expression arrays with respect to SNPs
Source: BMC Bioinformatics. 2007 Jul 30;8:276. doi: 10.1186/1471-2105-8-276 (PMC1959249; doi:10.1186/1471-2105-8-276)
Supplement: Additional file 13 — AffyMAPSDetector v1 source code. AffyMAPSDetector source code. [file 1471-2105-8-276-S13.zip › AffyMAPSDetector_v1_src/doc/jwf/WizardAdapter.html]

WizardAdapter


|  |  |  |  |
| --- | --- | --- | --- |
| **Overview** \| **Package** \| **Class** \| **Use** \| **Tree** \| **Deprecated** \| **Index** \| **Help** | | |  |
| **PREV CLASS**   **NEXT CLASS** | **FRAMES**    **NO FRAMES**     **All Classes** |  |  |
| SUMMARY: NESTED \| FIELD \| CONSTR \| METHOD | DETAIL: FIELD \| CONSTR \| METHOD |  |  |


---


## jwf Class WizardAdapter

```
java.lang.Object
  jwf.WizardAdapter
```

**All Implemented Interfaces:**: WizardListener

---

public abstract class **WizardAdapter** extends java.lang.Object implements WizardListener

This class provides means of abreviating work when using the WizardListener
allowing the developer to implement only the needed methods

**Author:**
:   rodrigomalara@users.sourceforge.net

---

**Constructor Summary**

`WizardAdapter()`
  
          Creates a new instance of WizardAdapter


|  |  |
| --- | --- |
| **Method Summary** | |
| `void` | `wizardCancelled(Wizard wizard)`             Called when the wizard is cancelled. |
| `void` | `wizardFinished(Wizard wizard)`             Called when the wizard finishes. |
| `void` | `wizardPanelChanged(Wizard wizard)`             Called when a new panel has been displayed in the wizard. |

**Methods inherited from class java.lang.Object**

`clone, equals, finalize, getClass, hashCode, notify, notifyAll, toString, wait, wait, wait`

**Constructor Detail**

### WizardAdapter

```
public WizardAdapter()
```

:   Creates a new instance of WizardAdapter


**Method Detail**

### wizardCancelled

```
public void wizardCancelled(Wizard wizard)
```

:   Called when the wizard is cancelled.

    :   **Specified by:**: `wizardCancelled` in interface `WizardListener`
    :   **Parameters:**: `wizard` - the wizard that was cancelled.

---


### wizardFinished

```
public void wizardFinished(Wizard wizard)
```

:   Called when the wizard finishes.

    :   **Specified by:**: `wizardFinished` in interface `WizardListener`
    :   **Parameters:**: `wizard` - the wizard that finished.

---


### wizardPanelChanged

```
public void wizardPanelChanged(Wizard wizard)
```

:   Called when a new panel has been displayed in the wizard.

    :   **Specified by:**: `wizardPanelChanged` in interface `WizardListener`
    :   **Parameters:**: `wizard` - the wizard that was updated


---


|  |  |  |  |
| --- | --- | --- | --- |
| **Overview** \| **Package** \| **Class** \| **Use** \| **Tree** \| **Deprecated** \| **Index** \| **Help** | | |  |
| **PREV CLASS**   **NEXT CLASS** | **FRAMES**    **NO FRAMES**     **All Classes** |  |  |
| SUMMARY: NESTED \| FIELD \| CONSTR \| METHOD | DETAIL: FIELD \| CONSTR \| METHOD |  |  |


---
